# Hydrogel- and Nanocomposite-Based Drug-Delivery Strategies in the Treatment of Vaginal Infections

**DOI:** 10.3390/polym16060775

**Published:** 2024-03-12

**Authors:** Renad AlAnsari, Bushra Hasan, G. Roshan Deen, Uwe Torsten

**Affiliations:** 1 Materials for Medicine Research Group, School of Medicine, The Royal College of Surgeons in Ireland (RCSI), Medical University of Bahrain, Busaiteen 228, Kingdom of Bahrain; 21200799@rcsi-mub.com (R.A.); 21200908@rcsi-mub.com (B.H.); utorsten@rcsi-mub.com (U.T.); 2 Department of Obstetrics and Gynecology, School of Medicine, The Royal College of Surgeons in Ireland (RCSI), Medical University of Bahrain, Busaiteen 228, Kingdom of Bahrain

**Keywords:** vaginal infections, biofilm, bacterial vaginosis, vaginal drug delivery, hydrogels

## Abstract

The reproductive health of women is governed by an optimal balance in the host–microbiota interaction. Depletion of the beneficial vaginal microflora caused by depletion of Lactobacillus species and increased proliferation of pathogens results in gynaecological infections. Among women of reproductive age, vaginal infections are increasingly prevalent. Attaining therapeutic efficacy using conventional formulations remains a challenge as vaginal fluids quickly remove or dilute the therapeutic formulations. Hydrogels have been widely exploited for targeted delivery of therapeutics directly into the vaginal mucus. With a careful choice of polymers (natural, synthetic, or semisynthetic), hydrogels with specific properties, such as stimuli responsiveness, antimicrobial, and muco-adhesiveness, can be tailored for higher therapeutic efficacy. In this review, the advances in hydrogel strategies for the treatment of vaginal infections are presented with emphasis on the types and properties that play a significant role in vaginal drug delivery systems.

## 1. Introduction

Vaginal infections are a global public health issue affecting worldwide up to 70% of women of reproductive age [1,2]. The symptoms or clinical manifestations are itching, irritation, abnormal vaginal discharge, and discomfort when urinating and during sexual activity. In some cases, the infections may be asymptomatic or show mild symptoms, The most common infections caused by bacteria, fungus, and protozoa are bacterial vaginosis (BV), vulvovaginal candidiasis (VVC), and trichomoniasis [3,4]. Bacterial vaginosis is caused by the species *Staphylococcus* and *Peptostreptococcus*, Enterobacteriaceae, *Gardnerella vaginalis*, and *Mycoplasma hominis*. Vulvovaginal candidiasis is caused by *Candida albicans*, *Candida tropicalis*, *Candida parapsilosos*, *Candida crusei*, *Candida glabrata*, *Candida stellatoidea*, and *Candida lusitaniae*. Trichomoniasis is caused by the human protozoan pathogen *Trichomonas vaginalis*.

**Figure 1:**
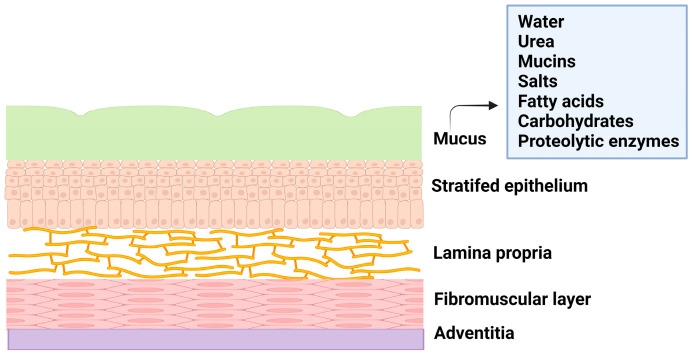
Illustration of layers of vagina tissue and mucus composition.

The female reproductive system is composed of the fallopian tubes, ovary, cervix, ectocervix, and vagina. The vagina is a distensible organ (~9 cm in length), which is characterized by a stratified epithelium, fibromuscular layer, lamina propria, and adventitia. The vagina is covered with cervical mucus which is composed of water (90%), urea, carbohydrates, mucins, fatty acids, proteolytic enzymes, and salts [5]. The consistency of cervical mucus and the thickness of the vaginal epithelium vary throughout the menstrual cycle and are regulated by hormones such as estrogen. The main functions of the mucus are to provide lubrication and protection against infections. The layers of vaginal tissue and compositions of mucus are illustrated in Figure 1.

Various factors, such as age, menstrual cycle, hormone level etc., influence the physiology of the vagina, and these factors affect the natural vaginal flora or microbiota and the amount of vaginal fluids. The *Lactobacillus* species is the predominant microorganism that protects the vagina from the invasion of pathogens by producing lactic acid that provides the acidic environment of the vagina. When this normal symbiotic mutual relationship becomes imbalanced, the natural vaginal flora is heavily disturbed, leading to the onset of vaginal infections that promote the colonization of pathogens.

**Figure 2:**
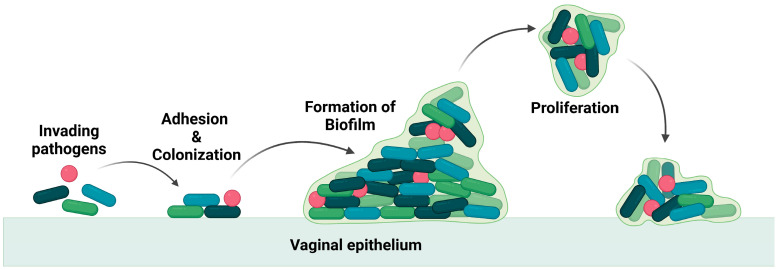
Illustration of invasion of pathogen and biofilm formation.

These pathogens thrive in the vagina by producing a biofilm that leads to the recurrence of the infection even after treatment [6,7], as the biofilm is an adaptation of the bacteria to resist any applied drugs and evade the immune system. The biofilm is composed of a group of pathogenic microbes adhered to one another on a surface that is, by extracellular polymeric substances, produced by the microbes. The formation of biofilm is illustrated in Figure 2. Biofilm is a physical barrier that limits the accessibility of drug molecules and enhances the thriving of pathogens. In this aspect, any drug formulation for vaginal delivery should not be harmful to the vaginal flora and its environment.

Vaginal infections, particularly bacterial vaginosis, are comparatively higher in non-pregnant women than in pregnant women and women at the age of 40 and above. This difference in the rate of infection is attributed to the imbalance of estrogen that affects the viability of the *Lactobacillus* species in the vagina.

## 2. Vaginal Infections and Treatment

Vaginal infections are a reproductive health issue, globally affecting women at their reproductive age, and the reported cases are of microbial origin. These include pathogenic bacteria, fungi, viruses, or parasites. The common types of vaginal infections are bacterial vaginosis (BV), vulva vaginal candidiasis (VVC) and trichomoniasis, human immunodeficiency virus (HIV) infections, and human papilloma virus (HPV) infections [1,2,3,4].

### 2.1. Bacterial Vaginosis (BV)

BV is the most prevalent infection globally, ranging from 23–29%, and the risk factors are sexual intercourse, douching, and poor hygiene. It is caused by an overgrowth of anaerobic bacteria and microaerophilic bacteria, such as *Gardnerella vaginalis*, *Autopodium vaginae*, *Bacteroides* spp., etc. The symptoms are thin white vaginal discharge with a fishy odour, irritation, and itchiness, and in general, the infection is diagnosed using the Nugent criteria, Amsel criteria, and Hay–Ison criteria [8]. The most common treatments for BV are antimicrobial formulations, such as oral metronidazole, oral clindamycin, oral tinidazole, metronidazole gel, clindamycin cream, and clindamycin ovules [9,10,11].

### 2.2. Vulva Vaginal Candidiasis (VVC)

VVC is primarily caused by the fungus *Candida albicans*, and it is estimated that nearly 75% of women will experience this infection at least once in their lifetime [12]. The symptoms of these infections are abnormal vaginal discharge, vaginal soreness, dyspareunia, and dysuria. Treatment of VVC is through oral and topical azole therapies, such as fluconazole, clotrimazole, miconazole, tioconazole, terconazole, and butoconazole [13,14,15].

### 2.3. Trichomoniasis

Trichomoniasis is a parasitic infection and is caused by a protozoan parasite, *Trichomonas vaginalis*. This is a nonviral sexually transmitted infection and, in 2016, has infected around 5.3% of women worldwide (the majority being asymptomatic). The symptoms of these infections are yellow–green vaginal discharge, lower abdominal pain, dysuria, and irritation in the vulva [16]. Infertility, poor pregnancy outcomes, and acquisition of sexually transmitted infections are associated with trichomoniasis [17]. The treatment is mainly through oral antibiotics, such as metronidazole and tinidazole. For complete eradication of the infection, topical formulations are not recommended due to the lower cure rate because of the poor residence time of the formulation on the vaginal tissues [18].

### 2.4. Human Immunodeficiency Viral Infection (HIV)

One of the most common viral sexually transmitted infections is HIV, with an estimate of about 38 million people being affected. Elevated risks are among intravenous drug users, sex workers, and the transgender community [19]. Symptoms of infections include liver dysfunctions, myalgias, swollen lymph nodes, tuberculosis, and AIDS. The currently available antiretroviral drugs are nucleoside or nucleotide reverse transcriptase inhibitors (tenofovir), non-nucleoside reverse transcriptase inhibitors (nevirapine), protease inhibitors (ritonavir, indinavir), integrase inhibitors (raltegravir), CCR5 antagonist (maraviroc), and fusion inhibitors (enfuvirtide) [20]. All of these drugs inhibit the various stages of the virus’ life cycle.

### 2.5. Human Papilloma Viral Infection (HPV) (Low Risk and High Risk)

**Figure 3:**
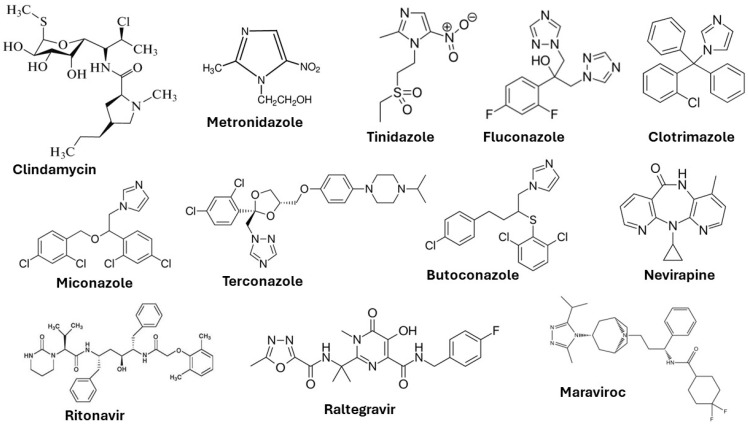
Chemical structures of drugs used to treat vaginal infections and sexually transmitted infections.

This viral infection occurs in the cervix and squamous epithelium (the basal layer) of the vagina. The virus is in the form of episomes in the basal layer and multiplies through the differentiation of epithelial cells [21]. Many patients in the initial stage of infection experience mild symptoms (development of genital warts) due to the production of antimicrobial peptides by the epithelial cells and the natural chemicals present in the vaginal mucus. Lasting HPV infections may result in the integration of the high risk HPV DNA into the host DNA and may lead to cervical, anogenital, and oropharyngeal cancer [22]. Vaccination and cryotherapy are the most effective approaches suggested by the World Health Organization (WHO) for the prevention of cervical cancer and the management of precancerous lesions [23]. The chemical structures of some important drugs used in the treatment of vaginal infections and sexually transmitted infections are shown in Figure 3.

In the management of vaginal infections, the oral administration of drugs and topical applications are some of the current therapeutic approaches. However, both forms of drug-delivery approaches suffer from numerous systemic adverse effects [24]. Oral administration of the antibiotic metronidazole causes nausea, insomnia, dizziness, and dry mouth. The long-term oral administration of this antibiotic may lead to leukopenia and neutropenia [25]. Oral administration of miconazole nitrate, an antifungal drug leads to thrombocytopenic purpura, a blood disorder [26]. Apart from these, oral administration of such drugs may have serious side effects in pregnant women and women suffering from gastrointestinal disorders.

Given the high exposure contact surface and dense vascularization, the vagina is an alternative site for local and systemic drug delivery. The main advantage is the avoidance of the acidic gastrointestinal environment, fewer side effects and bypassing of the hepatic first-phase effect. Several conventional formulations, in the form of capsules, creams, solutions, gels, and vaginal suppositories, are used in the treatment of vaginal infections. These pharmaceutical products for vaginal drug delivery also suffer from a few limitations, such as poor adhesion, short retention or residence time, and poor release of the drug [26,27]. To address these limitations, formulations based on hydrogels have been developed. Hydrogels are well-suited for controlled delivery of biologically active therapeutics owing to their biocompatibility, high porosity, and high water-retention properties.

In this manuscript, hydrogel-based formulations for the treatment of vaginal infections and important considerations on the choice of polymeric materials are reviewed. The criteria of hydrogels for vaginal drug delivery and the different types of hydrogels used are presented in this review.

## 3. Important Considerations on Hydrogel-Based Approaches to Vaginal Drug Delivery

Vaginal suppositories are often used in gynaecological treatments to locally deliver the drug with minimal systemic toxicity. Due to insufficient retention time of the drug in the suppository, the drug tends to leach out making this type of drug delivery less effective. As a result of this, frequent drug administration becomes necessary. Treatments that are ineffective could lead to various health-related issues, such as the development of bacterial resistance, inflammations, miscarriage, and infertility [28]. To overcome the issues, a variety of drug-delivery approaches have been developed for effective delivery of therapeutic agents to the vagina. In principle, an ideal vaginal drug-delivery system (VDDS), should be safe and release the drug in a sustained manner.

For improved vaginal drug delivery, formulations based on hydrogels have been developed. Hydrogels are three-dimensional crosslinked hydrophilic polymer networks that can absorb a large amount of water or physiological fluids without being dissolved [29]. The soft and rubbery nature of fully swollen hydrogels resembles the nature of many soft biological tissues, making these materials a good choice for drug-delivery applications. Depending on the nature of constituting polymers, hydrogels are classified as natural, synthetic, and hybrid (containing more than one type of polymer or component). The hydrogels are further classified into two main categories based on crosslinking, (i) chemically crosslinked hydrogels and (ii) physically crosslinked hydrogels.

**Figure 4:**
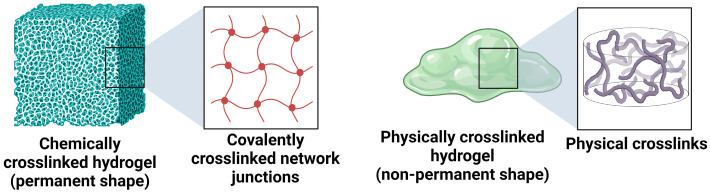
Illustration of chemically crosslinked and physically crosslinked hydrogels.

The chemically crosslinked hydrogels have permanent crosslink junctions through the formation of covalent bonds, while the physically crosslinked hydrogels have nonpermanent crosslink junctions that arise due to physical or secondary interactions. These interactions include polymer entanglements, ionic interactions, hydrogen bonding, and hydrophobic interactions [30]. The two types of hydrogels are illustrated in Figure 4.

For any gynaecological treatment, the most suitable hydrogel system would be the one with good shape-conforming properties. This property allows for a higher retention time of therapeutics in the vaginal mucosa and uniform distribution of the hydrogel over the area. Based on this criterion, chemically crosslinked hydrogels with defined and permanent shapes are not well-suited for vaginal drug delivery. Physically crosslinked hydrogels with nonpermanent or reversible crosslinks are the most suitable systems for VDDS. The reversible crosslink interactions can be modulated by in situ physical interactions, stereo complexation, inclusion complexation, and supramolecular interactions [31].

**Figure 5:**
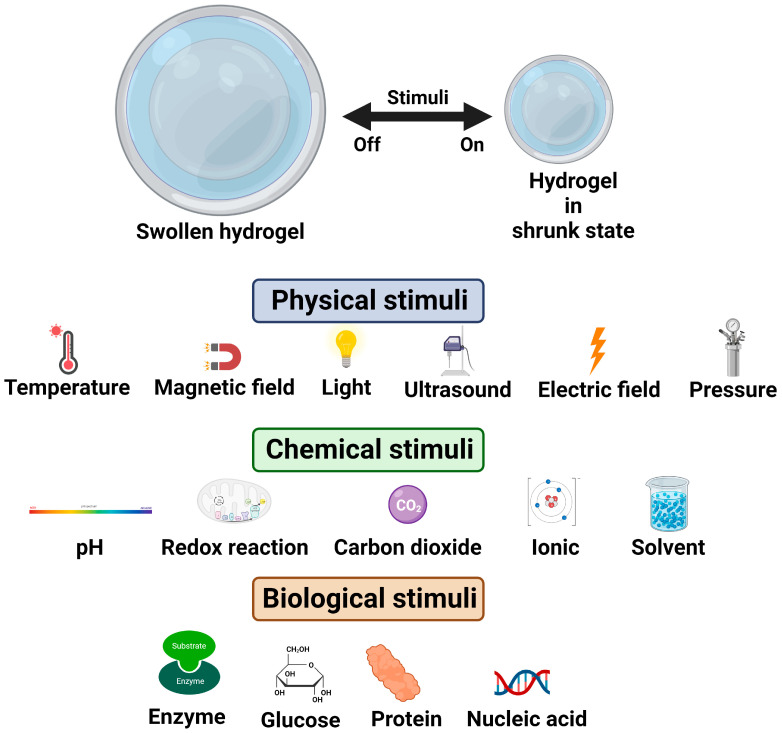
Illustration of stimuli-responsive hydrogels.

Stimuli-responsive hydrogels are an interesting class of hydrogels that respond to changes in external stimuli such as pH, temperature, ionic strength, surfactants, light intensity, biomolecules, and magnetic fields [32]. Upon the application of the stimuli, specific events such as in situ gel formation and drug release are evoked, and this property has made these materials very attractive for various types of drug-delivery systems. Further, hydrogels that respond to multiple stimuli or triggers have been developed to achieve better control over the responsive behaviour in target-specific drug-delivery systems [30,31,32]. The behaviour of stimuli-responsive hydrogels under various external stimuli is illustrated in Figure 5.

Stimuli-responsive dynamic hydrogels with reversible crosslink interactions and desirable rheological properties are well-suited for the vaginal drug delivery of therapeutic agents. The nature of the hydrogel matrix allows for the controlled encapsulation and administration of both hydrophobic and hydrophilic drugs, and greatly limits toxicity and side effects associated with the drug. In addition, the rheological properties should facilitate the convenient administration and even distribution of the gel (containing the therapeutics) on the mucosal tissue of the vagina.

### 3.1. Injectable Dynamic Hydrogels and Rheological Considerations

Injectable hydrogels are polymer systems that are liquid at room temperature but form a solid gel (through physical crosslinking) at the site of administration. Stimuli-responsive polymers undergo sol–gel transition in response to environmental triggers. Such injectable hydrogels containing therapeutics are most suited for intravaginal applications. However, the kinetics of gelation may be a disadvantage, i.e., if the gelation is very quick, the hydrogel can solidify within the syringe, or if the gelation is very slow, the therapeutics may be released prematurely [33]. The problem associated with gelation kinetics is overcome by injectable hydrogels that show dynamic properties.

Injectable dynamic hydrogels allow easy and reversible transition between the sol–gel states due to their unique rheological properties, such as shear thinning and self-healing. The clinical applications of such dynamic hydrogels are limitless, with new therapeutic platforms. The rheological modifications required for each clinical application need to be considered in relation to the constraints involved in injectability. In addition, thixotropic behaviour (transient recovery of viscosity after the flow of material had stopped) needs to be evaluated.

For intravaginal applications, which require high spreadability and strong mucoadhesion, the thixotropy evaluation provides valuable information, which determines the efficacy of the treatment. The thixotropic property is strongly dependent on the chemical functional groups of the polymers that are involved in physical crosslinking (sol–gel transition). This property provides important information on the time scale of gelation or erosion of material after the administration process [34].

The rheological properties of the hydrogels play an important role in the delivery of a wide range of drugs to the vagina for the treatment of vaginal infections. In general, the administrative process of injectable hydrogel formulations follows the following processes, viz. (i) compatibility of drugs and hydrogels without any reaction (any reaction between the components will lead to the loss of bioactivity of the drug), (ii) flow behaviour and injectability, and (iii) achieving the desired terminal function, such as strong mucoadhesion and sustained release of the drug.

For clinical applications, the injectable hydrogels are delivered through a needle or tube to the site of infection. The flow of the gel is directly dependent on the applied pressure, the viscosity of the formulation, and the geometry of the needle, catheter, or tube. It is critical to understand the rheological properties of vaginal formulations for high spreadability and mucoadhesion without erosion of the materials. The relationship between the viscosity of the hydrogel formulation and the required injection pressure is usually given by the steady-state flow models [35]. The viscosity profile follows a power–law for physically crosslinked hydrogels, as given below.

(1) $$\eta =K\;\gamma^{n-1}$$

where *K* is the consistency parameter, *γ* is the shear rate, and *n* is the shear-rate-thinning parameter. For a Newtonian fluid of constant viscosity as a function of shear rate, *n* = 1. A value of *n* < 1 indicates a shear-thinning fluid, and a value of *n* > 1 indicates a shear-thickening fluid.

Assuming a power–law relationship between the shear stress and shear rate, the injection pressure (*P*) for physically crosslinked hydrogels including polymer nanocomposites is given [36].

(2) $$P={\left(\frac{3\;n+1}{n}\right)}^{n}K{\left(\frac{Q}{\pi }\right)}^{n}\frac{2l}{R^{3n+1}}$$

where *Q* is the flow rate, *R* is the radius of the needle or tube, and *l* is the length of the needle or tube.

To account for significant yield stress, slip, and nonconstant geometries, alternative rheological models have been developed and validated. Designing new injectable vaginal formulations with tailored properties requires careful and rigorous characterisation of rheological properties, such as thixotropy, viscoelasticity, shear-thickening, shear-thinning, and yield.

### 3.2. Mass-Transport Considerations

The mass-transport process refers to the movement of drugs (also known as cargo) through the hydrogel matrix. Knowledge of this process is important for designing hydrogels as drug-delivery devices. The mass transport is influenced by the nature of the hydrogel and drugs, crosslink density, mesh size, porosity, and polymer architecture. The movement of drugs in the hydrogel depends on the hydrodynamic diameter of the drug (*R_D_*) and the mesh size of the hydrogel (*ξ*). Depending on the ratio between these two parameters, the release of the drug can be either diffusion dependent, erosion dependent, or both [37].

**Figure 6:**
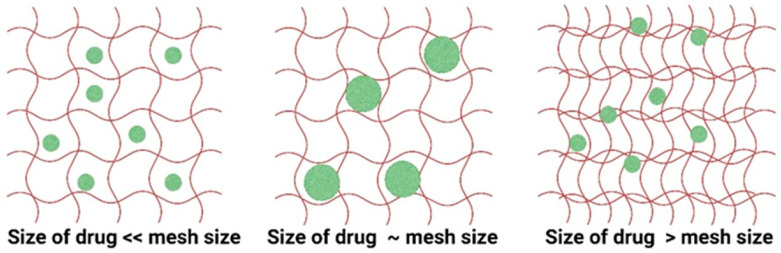
Illustration of transport of drugs from hydrogels based on the hydrodynamic radius of drugs and mesh size of the hydrogel matrix. The red lines are the polymer chains and the green spheres are the drug molecules.

For the diffusion-dependent mechanism, a short time frame of hours to days is involved, while for the erosion-dependent mechanism, the time frames are long, ranging from weeks to months. For the diffusion of a drug, three different cases can be identified: (i) if *R_D_* of the drug is < *ξ*, the drug diffuses freely in the volume of the gel leading to rapid exit or burst release, (ii) if *R_D_* ≅ *ξ*, the diffusion is slow and sustained, and (iii) if *R_D_* > *ξ*, the drug is immobilized in the hydrogel. The three different cases are illustrated in Figure 6.

Hydrogels for biomedical applications and drug delivery are often designed to degrade under physiological conditions. The degradation or erosion of the gel occurs through hydrolysis (regular and enzymatic) of chemical bonds of the hydrogel. The relationship between the drug size and mesh size of the hydrogel can be used to determine the kinetics of drug diffusion. For hydrogels for vaginal applications, the spreadability, mucoadhesiveness, and sustained release of the drug can be improved by varying the crosslink density of the hydrogel formulation.

### 3.3. Swelling Characteristics and Diffusion Parameters

Swelling in hydrogels occurs when a solvent or physiological fluid permeates and diffuses through the interstitial space within the hydrogel network. The network is directly related to the density of the polymer, the density of the crosslinker, and the mesh size. The swelling behaviour of hydrogel is affected by the nature of the polymers, the degree of crosslinking, and the nature of the swelling medium [38]. The equilibrium swelling ratio (*Q*) is defined as,

(3) $$Q=\frac{W_{s}-W_{d}}{W_{d}}$$

where *W_S_* and *W_d_* are the swollen and dry weight of the hydrogels, respectively.

The equilibrium swelling ratio provides information about the capacity of the hydrogel to absorb and retain water or physiological fluid. The swelling properties of hydrogels make them attractive for various drug-delivery systems. The other factors that affect the swelling are chemical composition, crosslink density, concentration of the drug, pH and ionic strength of the surrounding medium, temperature, etc. For vaginal drug delivery, adhesion, and cohesion of the gel formulation on the vaginal tissue needs to be considered. Adequate adhesion on the mucus promotes close contact between the hydrogel and target tissue, enhancing the drug-delivery efficiency. Cohesion is essential to maintain the integrity of the hydrogel and prevent drug leakage or migration [39].

## 4. Mechanism of Drug Release from Hydrogel Formulations and Mathematical Models

Depending on the composition of the hydrogel-based formulations for vaginal drug delivery, one or more of the following physical and chemical phenomena affect the drug-release kinetics [40]. These include (i) wetting of the surface of the drug-delivery device by the release medium, (ii) penetration of water or mucus into the device through pores, (iii) degradation of the formulation, (iv) leaching of the active drug from the hydrogel matrix, (v) swelling of hydrogel, (vi) osmotic and hydrostatic pressure, (vii) changes in microenvironmental conditions, (viii) chemical reactions, (x) convection due to hydrostatic pressure, and (xi) change in the geometry of the device. To describe the mechanism of drug release, other factors, such as enzymatic degradation, protein binding, and active and passive cellular drug uptake, also need to be considered.

The mechanism of drug release consists of the following phenomena, viz. exterior diffusion, interior diffusion, desorption, and chemical reactions. Exterior diffusion takes place when drug molecules diffuse from the surface of the hydrogel to the bulk of the liquid phase, and the rate of mass transfer (*G_A_*) is described by the following expression [41].

(4) $$G_{A}=k_{L}A\left(C_{AL1}-C_{AL2}\right)$$

where *k_L_* is the mass-transfer coefficient, *C_AL_*_1_ is the surface concentration of the drug, *C_AL_*_2_ is the bulk concentration of the drug, and *A* is the area of mass transfer. The mass-transfer coefficient is given as:

(5) $$k_{L}=\frac{{\left(D_{AB}\right)}_{L}}{\delta_{L}}$$

where (*D_AB_*)*_L_* is the drug-diffusion coefficient.

**Figure 7:**
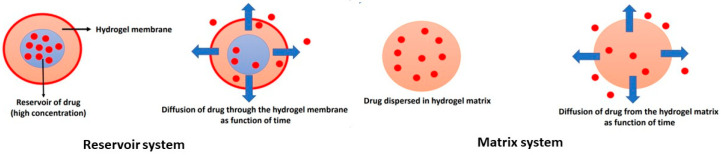
Illustration of major drug-delivery systems: the reservoir system and matrix or monolithic system. The drug molecules are represented by red spheres and the arrows illustrate the direction of diffusion.

The drug concentration is highest close to the surface of the hydrogel matrix and decreases with the depth of the gel. When the liquid in the bulk is stirred, the value of drug concentration is constant. Exterior diffusion controls the rate of drug release only in exceptional cases. The rate of drug release depends on interior phenomena or interior diffusion. The interior diffusion is based on Fick’s law of diffusion, and two types of systems are recognized, such as the reservoir system and the matrix or monolithic system, as shown in Figure 7.

In the reservoir system, the drug is surrounded by a polymer membrane, and diffusion of the drug through the membrane is the rate-limiting step. When the concentration of the drug is very high (saturated), a constant concentration gradient across the polymer membrane is achieved. The drug is absorbed from the inner bulk of the membrane and diffuses through the membrane. It is then desorbed from the membrane to the fluid that surrounds the reservoir device.

The drug release from the reservoir system across the polymer membrane is described by Fick’s law of diffusion [42].

(6) $$J=-D\frac{{dC}_{A}}{dx}$$

where *J* is the flux of the drug, *D* is the drug-diffusion coefficient, and *C_A_* is the concentration of the drug.

For a reservoir device with an initial concentration of the drug smaller than drug solubility, the drug concentration at the inner surface of the membrane decreases with time. For nonswelling or dissolving membranes with perfect sink conditions, the drug release is described by first-order kinetics, and the release rate is not dependent on the geometry of the device [43]. For systems with an initial drug concentration much larger than the solubility of the drug, the drug release follows zero-order kinetics and is independent of the geometry of the drug-delivery device.

In a matrix system, the drug is equally dispersed or dissolved in the hydrogel matrix with a decreasing release rate with time. The nonsteady state diffusion of a drug in a one-dimensional slab is described by Fick’s second law of diffusion [44].

(7) $$J=\frac{{dC}_{A}}{dx}=D\frac{d^{2}C_{A}}{{dx}^{2}}\;$$

For monolithic devices, the geometry of the system strongly affects the drug release profile and different analytic expressions have been introduced for various geometries such as thin films with negligible edge effects, spherical, and cylindrical [45]. Fick’s law equations cannot be solved analytically when more complex geometries or nonconstant drug diffusivities are incorporated into the mathematical models. In the case of hydrogels, the diffusivity of the drug is affected by the degree of swelling and crosslinking density of the material or formulation.

**Figure 8:**
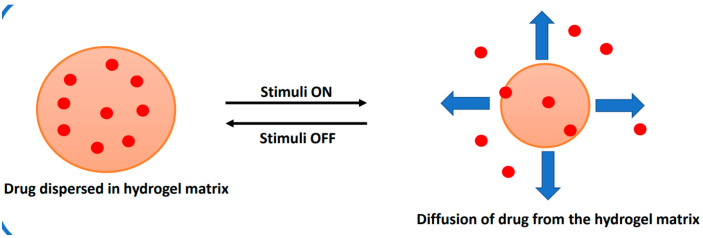
Illustration of drug diffusion from a stimuli-responsive drug-delivery system.

In the case of stimuli-responsive drug delivery, the drug diffuses from the hydrogel matrix in response to changes in external stimuli, such as pH, temperature, pressure, biomolecules, light, ionic strength, and magnetic field, as illustrated in Figure 8.

## 5. Types of Hydrogel Formulations as Vaginal Drug-Delivery Systems

### 5.1. timuli-Responsive Hydrogels

For gynaecological treatments of vaginal infections, the vaginal drug-delivery route is the most preferred method, as it avoids first-pass metabolism and provides effective delivery of therapeutics at the site of infection. The vaginal secretions along with the self-cleansing actions of the vagina could potentially dilute the formulation, leading to less efficacy of the therapeutics. Among several polymeric materials, polymers with mucoadhesive and thermos-responsive properties have attracted great attention for vaginal drug delivery. As discussed in the earlier section, stimuli-responsive polymers and hydrogels are well-suited to overcome the limitations of other drug-delivery systems.

**Figure 9:**
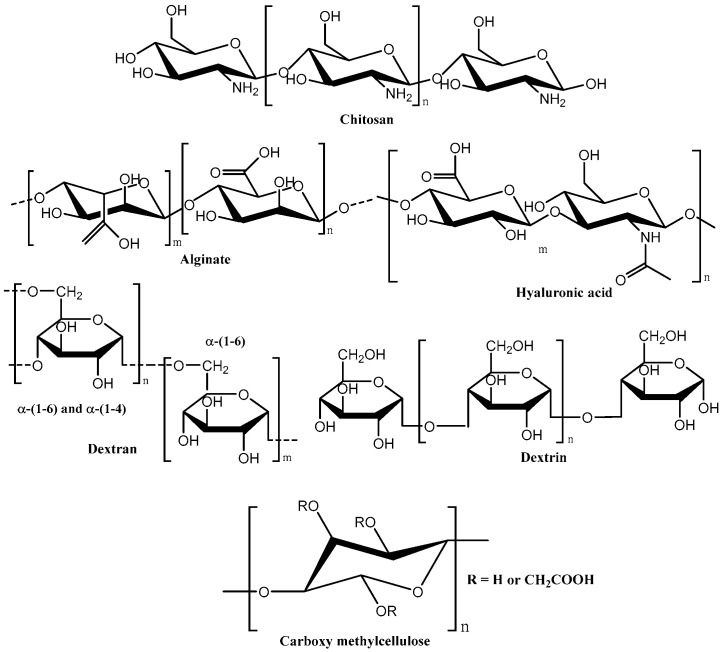
Chemical structure of some important natural polymers used in hydrogels for the treatment of vaginal infections.

These materials sharply respond to slight changes in external stimuli due to changes in interactions between the polymer chains leading to changes in macroscopic properties. The molecular structures of a few important natural polymers [30,31,32,33].used in hydrogels for gynaecological treatment are shown in Figure 9.

### 5.2. Temperature-Responsive Hydrogels (Thermogelling Systems)

Hydrogels that undergo sol–gel transition in response to changes in external temperature are classified as temperature-responsive hydrogels or thermogelling hydrogels. Thermosensitive hydrogels based on natural and synthetic polymers with thermosensitive properties are widely used in vaginal drug delivery of therapeutics.

For formulations that are of low viscosity, they can be applied in the form of aerosols using a propellant such as propane–butane in the ratio 80:20 *v*/*v*. Thermogelling aerosol gels containing Carbopol, triblock polymers such as Pluronic F68, and poloxamer 407 have been used in the treatment of cervical erosion and vaginitis [46]. The formation of aerogel foam improved the penetration efficiency of the formulation increasing the strength of mucoadhesion of the gel, leading to initial rapid release of the drug followed by sustained release.

Chitosan-based hydrogels in combination with β-glycerol phosphate have been used widely in gynaecological antimicrobial treatment [47]. Chitosan is a polysaccharide obtained from chitin found in the shells of crustaceans and is composed of β(1,4)-linked glucosamine and *N*-acetyl glucosamine subunits. This natural polymer is widely used in biomedical applications due to its excellent properties, such as biocompatibility, biodegradability, and antimicrobial effects. The thermoresponsive gelation of chitosan with β-glycerol phosphate arises due to increased hydrophobic interactions (due to neutralization of chitosan) in response to changes in external temperature. Thermoresponsive hydrogels based on chitosan and β-glycerol phosphate, containing the drug auranofin, have shown positive effects in the treatment of vaginal infections caused by *Trichomonas vaginalis*.

Hydrogels based on chitosan (high concentration) and poloxamer 407 have been developed for the treatment of vaginitis. This highly viscous formulation provided high mucoadhesion, providing sustained release of the drug, thereby decreasing the drug administration frequency. Formulations of high viscosity improve the mechanical properties of the hydrogel and these are features that are required for effective vaginal gels as it allows for localization of antimicrobial drugs. Upon administration, the high viscosity of the chitosan promoted intermolecular interactions through polymer entanglements and dispersion forces which leads to the in situ formation of gels with good compressibility, cohesiveness, elasticity, and adhesiveness.

Chitosan on its own forms a gel under acidic pH conditions (pH = 5) with non-Newtonian fluid behaviour. Chitosan-based hydrogels containing extracts of the plant *Mitracarpus frigidus* (Rubiaceae family) have been used in the treatment of vulvovaginal candidiasis. The active chemical ingredients in the plant extract, such as kaempferol, kaempferol-*O*-rutenoside, methyl ursolate, ursolic acid, scopoletin, and psychorubin, have been recently identified as the main ingredients with antimicrobial properties [48]. A chitosan-based formulation containing an essential oil from the plant *Pelarognium graveoleus* (geraneus oil) has also been effective in the treatment of vulvovaginal candidiasis [46,47,48].

Thermogelling hydrogels based on hydroxy propyl methylcellulose (HPMC), and poloxamer 407, containing the drug tinidazole have shown promising application in the treatment of bacterial vaginosis. The improved mechanical property of the gel allowed good spreadability and high mucoadhesion, which provided longer residence time for the drug with sustained release. However, this formulation promoted little irritation as confirmed by the chorioallantoic membrane test (Hen’s Egg Test). Hydrogel formulation based on a mixture of polymers, such as Pluronic F127, Pluronic F68 and polycarbophil, containing the drug clotrimazole was effective in the treatment of vulvovaginal candidiasis (VVC). The combination of different polymers promoted good spreadability, mucoadhesion, and gelation at various temperatures due to the different gelation points of the individual polymers. An increase in the concentration of polycarbophil in the formulation enhanced the mucoadhesive properties of the gels through various physical interactions between the carboxyl groups (–COOH) of polycarbophil and glycoproteins present on the mucous of the vaginal epithelium [49].

A thermogelling formulation based on Pluronic F47 loaded with amphotericin-B was effective in the treatment of VVC due to enhanced permeation of the drug [50]. The formation of polymeric micelles at the sol–gel temperature increased the viscosity, allowing for the formation of a porous gel. The encapsulation of the drug within the micelle favoured local administration and sustained release. The formulation had low minimum inhibitory values (MIC) against many fungi, including *Candida albicans*, *Candida parapsilosis*, and *Candida glabrata*. Thermogelling formulations containing nanoparticles have also been developed for the treatment of VVC. Tenofovir-loaded chitosan nanoparticles containing formulations released the drug in two phases with good residence time onto the vaginal mucus. Pluronic-based formulations with natural polymers have been widely used for gynaecological therapies for the controlled delivery of antimicrobial drugs, such as metronidazole, amoxicillin, amphotericin, and clotrimazole [48,49,50,51]. Some important types of temperature-responsive formulations and gelation temperatures for vaginal drug delivery are given in Table 1.

### 5.3. pH-Responsive Hydrogels

Hydrogels that respond reversibly to slight variations in external pH conditions have been used in various drug-delivery systems. The normal pH range of the vagina is between 3.8 to 5.0, and variation in pH can significantly affect the vaginal drug-delivery system. Bacterial infections are one factor that can significantly affect the pH of the vagina. Apart from the commercially available vaginal films and membranes, new pH-responsive hydrogels and organogels have been developed. A pH-responsive multicomponent formulation based on hydrogels and organogels containing the drug Tenofovir has been developed for prophylaxis of HIV infection [51].

The heteropolysaccharide pectin in the hydrogel component provides a porous structure with a dense polymeric matrix that aids in increasing the mucoadhesive property of the formulation and rapid release of the drug. The esterification reaction of carboxylic acid groups of pectin and glycoproteins present in the vaginal mucous leads to the formation of more hydrogen bonds. This type of intermolecular structure is responsible for the formation of a dense polymer matrix in response to external pH changes.

Nontoxic and highly flexible pH-responsive vaginal films based on chitosan citrate and polymethylmethacrylate have been developed by layer-by-layer techniques for improved pH-responsive properties. The amount of crosslinker present in the film modulated the swelling behaviour and water penetration. The release of the drug Tenofovir was rapid in the simulated vaginal fluid. Polyurethane-based hydrogels and polyurethane membranes in the form of a reservoir intravaginal ring have also been developed for the on-demand delivery of small interfering RNA in the treatment of HIV infections.

Hydrogel nanofibers containing polyurethane and 1,4-bis (2-hydroxyethyl) piperazine showed good pH-responsive properties due to the protonation of amino nitrogen of piperazine under acidic pH conditions [52]. To prevent the proliferation and migration of virions in the vagina, a pH-responsive biologically inspired synthetic mucin-like polymer system was developed. This polymer system based on phenylboronic acid and salicyl hydroxamic acid, showed both shear-thinning and shear-thickening behaviour. The interaction of polymer components and the vaginal mucin resulted in good mucoadhesion and impeded the migration of virion in the vagina. Some important types of pH-responsive hydrogel formulations for vaginal drug delivery are given in Table 2.

### 5.4. Ion-Responsive Hydrogels

Polymers that form physically crosslinked gels in the presence of certain ions are called ion-responsive or ion-sensitive polymers. The presence of charged chemical functional groups in the polymer facilitates the reversible crosslinking process. The vaginal fluid contains ions of sodium (Na^+^), potassium (K^+^), calcium (Ca^2+^), and chloride (Cl^−^). These ions can crosslink in situ with certain types of polymers, such as alginate, chitosan, and gellan gum (polysaccharides), and form a solid gel. These types of materials are used in VDDS to improve bioadhesion and drug efficacy. Gellan gum is a natural polysaccharide (anionic) with repeating units of α-L-rhamnose, β-D-glucose, and β-D-glucoronate. The mole ratio of the three glucose units is 1:2:1. The carboxylic groups of the β-D-glucoronate are responsible for electrostatic interactions with the ions, leading to the formation of a gel [53].

Vaginal gels based on chitosan (1% *w*/*v*) and gellan gum (1% *w*/*v*), containing the model drug clindamycin, are used in the treatment of BV. This type of gel formulation has good adhesion on the vaginal mucous and has improved retention of the drug. Gellan gum and sodium carboxy methylcellulose-based formulations have been successful in the delivery of secnidazole for the treatment of vaginal trichomoniasis. The ion-responsive gelation process after administration was enhanced by the addition of calcium chloride (CaCl_2_) and sodium citrate (Na_3_C_6_H_5_O_7_) in the formulation [54].

Ion-responsive hydrogels based on cationic copolymers of methyl methacrylate, ethyl acrylate, and a low content of methacrylic acid ester with quaternary ammonium groups (Eudragit^®^ RS-100) in the form of nanocapsules have been used to deliver the drug indole-3-carbinol for the treatment of vaginal trichomoniasis [55]. Ionic crosslinking through thiol functional groups (-SH) has also been exploited in hydrogel formulations for gynaecological treatments. A multicomponent system consisting of eight-arm poly(ethylene glycol) with terminal thiol groups and dendrimer based on 4-poly(amidoamine) functionalized with thiopyridyl end groups has been used in the delivery of the antibiotic amoxicillin. The sol–gel transition is facilitated by intramolecular disulphide crosslinks that provide a long residence time for therapeutics. Interestingly, this hydrogel formulation has been found to be suited for intravaginal treatment in pregnant women. This is because the gel formed due to disulphide crosslinks of the dendrimer prevents diffusion across the membrane of the foetus [56].

The synthetic polymer Carbopol has also been used as an ion-responsive material in gynaecological hydrogel formulation in the treatment of bacterial vaginosis. Carbopol is a high molar mass poly(acrylic acid) which is crosslinked with pentaerythritol allyl esters. The sol–gel transition is modulated by the change in the external pH conditions and the presence of ions. Star-shaped polymers, polyethylene glycol, thiolated carboxymethyl hyaluronic acid, and poly(ethylene glycol)-bis bromoacrylate have all been used in VDDS as ion-responsive systems, some in the form of dry films and vaginal in situ gel [57]. Some important ion-responsive hydrogel formulations for vaginal drug-delivery systems are given in Table 3.

### 5.5. Multi-Stimuli-Responsive Hydrogels

Polymers that respond reversibly to more than one external stimulus are termed multi-stimuli-responsive polymers, and these materials open new avenues for smart and targeted vaginal drug delivery. Multi-stimuli-responsive polymers are synthesized either by combining two or more polymers with the desired effects or by incorporating the required functional groups in the same molecule. A critical balance of hydrophobic and hydrophilic groups is necessary to achieve the multi-stimuli-responsive effect. When two polymers are combined for multiple sensitivity, the effect can be very synergistic. Natural polymers in combination with synthetic polymers provide better multistimuli properties, and such systems are widely used in drug-delivery systems.

Chitosan in combination with poly(*N*-isopropyl acrylamide) provides pH and temperature-responsive properties. By varying the composition of the two polymers the properties can be easily tuned for intended applications. Ion and temperature-responsive physically crosslinked gels based on Pluronic PF-127/68 and gellan gum have been developed for the vaginal delivery of clindamycin. This system, when combined with carrageenan, improved the mucoadhesive property and prolonged the vaginal residence time of the drug for up to 9 h.

Temperature- and pH-responsive gels based on sodium alginate and PNIPAM with a high swelling ratio have been developed for sustained release of the antibiotic oxytetracycline. The high swelling of the gel is due to ionic interactions between the carboxylate groups of sodium alginate. Ketoconazole is an antifungal drug used to treat vaginal infections. This drug, however, has poor water solubility, and the solubility can be improved through the inclusion of complexes with cyclodextrins.

Chitosan and gellan gum-based hydrogel encapsulated with ketoconazole and β-cyclodextrin was effective in the delivery of the drug. The gel was developed in the form of flakes for better penetration and sustained release of the drug over 6 h through the vaginal mucosa. Gels consisting of pH and temperature-sensitive chitosan-graft-PNIPAM and polyvinyl alcohol have been developed for the safe delivery of voriconazole to the vaginal mucosa. The presence of PVA in the gel provided non-Newtonian properties and increased the viscosity for better adhesion over the mucus of the vagina.

Temperature- and enzyme-responsive organogels based on hyaluronic acid and palm oil have been developed for the delivery of the antiretroviral drug maraviroc. Organogels have a hydrophobic interior that is helpful for encapsulating hydrophobic drugs like maraviroc. As for the release of the drug in the presence of the enzyme hyaluronidase, the release of the drug was more than 2.5-fold due to the enzymatic backdown of the glucuronic acid units of the polymer. The organogels gel preserves the viability of the vaginal microbiome *Lactobacillus crispatus,* making this gel a good vehicle for mucoadhesion for vaginal microbicides [58].

pH-responsive osmotic pump tablets for vaginal delivery of the antiretroviral drug IQP-0528 have been developed based on cellulose acetate phthalate. The polymer core undergoes swelling when in contact with the simulated vaginal fluid and extrudes into the vaginal canal. When in contact with simulated seminal fluid, the coating of the tablet dissolved slowly giving a burst release of the encapsulated drug. In comparison to conventional vaginal tablets, the osmotic pump tablets provided a stable drug concentration in the vaginal mucosa and fluid for a period of up to 10 days, allowing sustained release of vaginal microbicides.

pH- and temperature-responsive liposomes based on methoxy polyethylene glycol 2000-hydrazone-cholesteryl hemi succinate have been developed for the vaginal administration of arctigenin in the form of liposomal gel or lipogel. The sol–gel transition of the gel was induced by temperature changes and drug release was achieved due to polymer degradation under acidic pH conditions. During vaginal delivery of arctigenin, the poloxamers of the liposomes greatly reduced its toxicity and improved the drug-release efficacy. Some important multi-stimuli-responsive hydrogel formulations for vaginal drug-delivery systems are given in Table 4.

### 5.6. Liquid Crystalline Hydrogels

Hydrogels based on liquid-crystal systems have been explored in the vaginal administration of therapeutics due to their low viscosity and ability to form in situ crystalline phases in liquids. When in contact with the vaginal mucous, the polymer undergoes phase transition with changes in flow behaviour due to the formation of self-assembled structures. A liquid-crystal system containing the extract of *Syngonanthus nitens* (grass-like plant species) has been studied in the treatment of vaginal infections caused by *Candida krusei* and *Candida albicans.* The extract-loaded system, on contact with vaginal mucous, forms semisolid crystalline structures with increased cell permeability. This promotes the uptake of the extract or drug leading to inhibition of the vaginal pathogens. Liquid gels based on phytantriol that forms liquid crystalline gels even in small amounts of vaginal fluid have been developed for the sustained delivery of sinomenine hydrochloride. The formation of a cubic liquid crystalline structure adheres strongly to the vaginal mucosa and prevents vaginal cleansing action. This type of material is being investigated for vaginal topical applications due to its mild irritation and inflammatory properties.

Vaginal mucoadhesive systems based on liquid-crystal precursors containing poloxamers, such as Carbopol974P and polycarbophil, have been developed for the treatment of vulvovaginal candidiasis. These materials had strong mucoadhesion force of up to 13 mN, with MIC values in the range of 31.2–62.5 μg mL^−1^. Mucoadhesive gels containing P407 and the drug hypericin have been investigated for therapeutic potential in the treatment of vaginal bacterial infections and gynaecological cancer. This gel, even after dilution, exhibited very high mucoadhesion to the vagina with a force of 26 mN and showed high cell viability [59]. Some important liquid-crystal hydrogel formulations for vaginal drug-delivery systems are given in Table 5.

### 5.7. Astrodimer Gel and Metronidazole in the Treatment of Bacterial Vaginosis

The astrodimer gel is a dendrimer-based mucoadhesive gel that was used in a phase 3, placebo-controlled, double-blinded study to compare the efficacy and safety of a placebo against the astrodimer 1% gel. The study comprised administering 5 g of the gel vaginally for 7 days. The endpoint of the study was indicated by the absence of vaginal discharge, less than 20% clue cells, and a negative whiff test, which was related to the absence of a bacterial vaginosis (BV) infection. The adverse effects that were reported during the study were considered mild to moderate, as well as tolerable, which resulted in the conclusion that supported the statement regarding the astrodimer gel being safe, effective, and beneficial towards the treatment of BV infections.

Metronidazole (MZT) is known to be a standard treatment for BV infections. The limitations of this treatment include potential toxicity and the ability of the MZT to restrict the growth of normal, beneficial flora in the vagina. Shaaban et al. conducted a randomized controlled trial (RCT) that investigated the analysis of efficacy in the use of an in situ formulation of the MZT vaginal gel once every day and the use of a conventional formulation of the gel twice every day [60].

The results were collected at the end of the first and fourth weeks to analyze the cure rates. In regards to the in situ gel, in the first week, there was a 74.5% cure rate, and in the fourth week, a 66.7% cure rate was seen. When the results of the conventionally administered MTZ gel were analyzed, it was observed that in the first week, a 63% cure rate was seen, and in the fourth week, 40% of the cases that involved BV infections were cured. At the end of this RCT, it was concluded that the in situ MTZ gel was more effective when it came to identifying a persistent, reliable cure in the treatment of BV. The reasons for this could be due to the demonstrated muco-adhesive properties and the ability of the thermos-responsive formulation to convert the solution into a gel, which would allow it to have an increased ability to stay in contact with the vaginal surface.

### 5.8. Nanocarriers: Liposomes, Polymer Nanoparticles, Fibers, and Gels

In view of their appealing properties, such as controlled release, precise targeting, and mucus adhesion, nanomaterials, such as liposomes and polymeric nanoparticles, have been used in VDDS. Azithromycin liposomes with different bilayer elasticities have been developed by Vanić et al. for the treatment of cervicovaginal infections [61]. The rigid bilayer properties allowed the slow release of azithromycin and prevented the formation of biofilms with half-maximal inhibitory concentration (IC_50_). A significant inhibitory effect on biofilms was observed with deformable propylene glycol liposomes that contained propylene glycol and monoacyl phosphatidylcholine. Pregnant women are highly vulnerable to vaginal infections caused by the human simplex virus (HSV).

This is due to reduced activities of T-helper-cell type 1 to protect the growing foetus [62]. The current antimicrobial therapy is not beneficial for controlling the infection due to resistance from the pathogen. Mucoadhesive liposomes decorated with chitosan and encapsulated with resveratrol have been developed to improve the bioavailability of the drug in the treatment of vaginal inflammation and infections. Mucin-binding studies of these liposomes in both normal (healthy) vaginal conditions and infected vaginal conditions showed enhanced mucin-binding properties at low chitosan concentrations [63].

Polymeric nanoparticles have been widely investigated as VDDS to enhance the residence time and delivery of therapeutics in the vagina. Chitosan is a cationic natural polymer that can easily form polyelectrolyte assemblies, and this feature has been widely exploited in various drug-delivery systems. Polymeric nanoparticles based on PLGA with modified surfaces containing chitosan were developed to deliver clotrimazole in the vagina. The mucoadhesion was enhanced due to the presence of chitosan on the surface of the polymeric nanoparticles which allowed for the formation of polyelectrolyte complexes when in contact with the mucin of the vaginal mucus.

The modified polymeric nanoparticles exhibited a higher biphasic drug-release profile for up to 18 days and antibacterial and antifungal effects. Chitosan-based nanoparticles containing miconazole nitrate have been effective in reducing the colony-forming unit levels, and cellular infiltrate. This effect was achieved with a significantly lower concentration of the drug relative to the conventional formulation [64].

Chitosan nanoparticles containing econazole were effective in the sustained release of the drug relative to the free drug solutions. The formulation was noncytotoxic and exhibited a low minimum inhibitory concentration value against *Candida albicans* [65]. The nanoparticles also showed an excellent time-to-kill profile for the complete eradication of *C. albicans* culture in just three days. The results suggest that formulations containing drug-loaded chitosan nanoparticles could be effective for the local treatment of vaginal candidiasis. Patient compliance and enhanced therapeutic delivery can be improved in the future by incorporating polymeric nanoparticles in films or spray gels [66].

Gels containing liposomes (pectin-coated sertaconazole-loaded liposomes) were more effective in reducing drug penetration through vaginal mucosa than the conventional gel without the pectin coating. Pectin-coated liposomes showed higher adsorption of mucin with more retained in the vaginal tissues [67,68]. Similarly, a mucoadhesive gel containing benzylamide hydrochloride-loaded liposomes exhibited high mucoadhesion. The polymers present in the liposome gel (lipogel), such as hydroxypropyl methylcellulose and Carbopol^®^974P, further enhanced the mucoadhesion properties of the formulation.

Polymeric films containing therapeutics have been widely explored in the treatment of vaginal infections. Polymer films with the desired properties and containing pharmaceuticals can be achieved easily. Polymer films of chitosan and poly(2-ethyl-2-oxazoline) containing ciprofloxacin have been developed for the treatment of vaginal infections. Polymer membranes containing alginate, chitosan, and metronidazole adhered strongly to the vaginal tissue and were effective against *E. coli*, *S. aureus*, and *G. vaginalis* [69]. Antimicrobial vaginal films of thiolated gellan gum loaded with metronidazole showed strong mucoadhesion and prolonged residence time of the drug.

The composition of the polymer greatly affects the adhesive properties of the films due to variations in the swelling index in response to the composition of the vaginal mucus. The bilayer vaginal films based on ethyl cellulose, tragacanth gum, and xanthan gum containing tenofovir have been explored for pre-exposure prophylaxis of HIV in women [70]. Films containing a high concentration of xanthan gum were highly mucoadhesive and nontoxic with a long drug-residence time. The choice of polymers is an important criterion for vaginal film platforms and affects the efficacy of the loaded therapeutics.

Polymers with strong mucoadhesive properties have also been exploited in VDDS, as they improve the adhesion of the formulation and retain the therapeutics for a longer time. The healthy vaginal mucosa consists of a squamous epithelium that is stratified and multilayered. In response to hormonal and environmental conditions, the epithelium undergoes changes throughout the lifetime of a woman. The structure of the epithelium also varies according to the age of the individual [71].

The stratum corneum of the vagina is enriched with mucins that are mainly composed of water, proteins, and lipids and forms a gel-like lining on the mucosal tissues. The delivery of therapeutics in the vagina by means of non-mucoadhesive formulations remains a challenge due to poor adhesion, nonuniform surface coverage, and poor adsorption. Mucoadhesive polymers interact with the vaginal mucosa to improve the adhesion and delivery of therapeutics.

A mucoadhesive formulation based on microgels containing polycarbophil and miconazole nitrate-loaded solid lipid particles exhibited high mucoadhesive strength and sustained drug release profile. Apart from being a nonirritant formulation, it was more effective in the eradication of C. albicans than the commercially available Daktarin^®^ cream [72]. Nanocapsules containing Eudragil^®^ RS100 encapsulated with indole-3-carbinol were effective in controlling the reactive oxygen species through its anti-inflammatory effect. The addition of gellan gum to the formulation resulted in higher mucoadhesion, and it is considered a safe alternative therapy for vaginal trichomoniasis [53].

Nanoemulsions containing oxiconazole nitrate, carboxymethyl cellulose, xanthan gum, and Carbopol 934 adhered strongly to the mucus and were effective in the penetration of the fungal cell wall. The formulation exhibited long drug resident time and controlled release profile and was effective against vaginal candidiasis [73]. The different types of mucoadhesive polymeric materials and formulations are present in Table 6.

## 6. Conclusions

To overcome the issues that arise with the most common vaginal infections, such as bacterial vaginosis and candidiasis, drug delivery through the vagina has established itself as a promising site to administer drugs through a muco-adhesive system which has been used for systemic and local effects. It involves the addition of mucoadhesive polymers such as chitosan, gellan gum, HPMC, and xanthan gum, these polymers have shown great biocompatibility, nontoxic effects, and are safe. This system can be applied in a variety of forms, including vaginal tablets, gels, films, and microparticles.

Stimuli-responsive systems have been of great interest in this field. The associated features of these systems allow them to have maximal surface coverage, as they are administered as a low viscosity liquid over the mucosal area and transform into in situ gels in response to environmental stimuli. Their mode of administration allows a prolonged formulation residence time in the lumen of the vagina, which plays a significant role in patient adherence and compliance. The literature has shown that pH-responsive systems and thermal-responsive systems play a role in improving efficacy, as the drug is applied locally through the vaginal route. Ion-responsive systems are considered a relatively new branch of stimuli-responsive systems, as only a limited number of studies have been conducted on this drug-delivery system. The safety concerns and clinical applications need to be investigated to further observe and explore the roles of this system and the polymers involved.

Multiresponsive systems which have a characteristic feature of undergoing chemical and/or physical changes as they respond to environmental stimuli have gained considerable interest and attention as drug-delivery systems. It involves the synthesis of formulations that are not considered feasible and can be challenging. An obstacle that is faced is the variety of vaginal environments during the life span of a woman which needs to be further researched to allow for a personalized regimen to provide better treatment for vaginal infections and disorders. The effectiveness and tolerability of formulations for these drug-delivery systems require further experimental studies to be able to achieve the goal of treating the various vaginal conditions at all stages. 

## Figures and Tables

**Table 1:** Some important temperature-responsive hydrogel formulations for vaginal drug delivery [19,20,21].

Formulation	Polymer	Gelation Temperature (°C)	Infection
Foam aerosol (thermal gelling) with silver nanoparticles	Pluronics, Carbopol	35.7	Vaginitis
In situ thermal gel with benzydamine hydrochloride	Poloxamer, chitosan	31.8	Vaginitis
Vaginal gel with clotrimazole	Pluronics F127, F68 and polycarbophil	31	Candidiasis
Thermoreversible gel with amphotericin	Pluronic P407	32	Candidiasis
Vaginal suppository with miconazole nitrate	Pluronics F188, P407 and HPMC		Candidiasis
Nanocomposite gel with auranofin	Chitosan, Pluronic F127, P407	32 °C	Trichomoniasis
Vaginal gel with tenofovir	Pluronics F127, Chitosan	26 °C	HIV
Blended hydrogel with curcumin	Pluronic P407, Chitosan, HPMC	32.8	Vaginal mucosal inflammation, HPV

**Table 2:** Some important pH-responsive formulations for vaginal drug delivery.

Formulation	Polymer	pH	Infection
Bigels with tenofovir	Pectin	4–12	HIV
Vaginal film with tenofovir	Chitosan citrate, Eudragit S100	2–3	HIV
Intravaginal ring (hydrogel) with siRNA	PEG, polyurethane	4–7	HIV
Intravaginal ring (with polymer membrane) with SiRNA	PEG, phenyl isocyanate, 1,4-bis(2-hyroxy ethyl piperazine)	4.5–7.0	HIV
Synthetic mucin like	Phenylboronic acid, salicylhydroxamic acid, crosslinked polymers	4.8–7.0	HIV

**Table 3:** Some important ion-responsive hydrogel formulations for vaginal drug delivery.

Formulation	Polymer	Drug	Infection
Vaginal gel	Gellan gum	Clindamycin	BV
Vaginal gel/solution	Gellan gum, HPMC	Clindamycin	Vaginitis
Vaginal gel	Gellan gum	Secnidazole	Trichomoniasis

**Table 4:** Some important multi-stimuli-responsive hydrogel formulations for vaginal drug delivery.

Formulation	Polymer	Stimuli	Infection
Gel with clindamycin	Gellan gum, pluronics	Temperature and ions	BV
Hydrogel with oxytetracycline	Sodium alginate, NIPAM	Temperature and pH	BV
In situ gel with polymer flakes and ketoconazole	Pluronic Pf127, chitosan, gellan gum	Temperature and ions	VVD
In situ gel with amphotericin	Copolymers	Temperature and pH	VVD
In situ gel with voriconazole	Grafted copolymers of NIPAM and PVA	Temperature and pH	BV and VVD
Organogel (palm oil-based) with maraviroc	Hyaluronic acid	Temperature and enzyme	HIV
Vaginal osmotic pump with antiretroviral drugs	Carbopol	Osmotic pressure and pH	HIV
Liposome gel with acrtigenin	Pluronics P407, PF188	Temperature and pH	VVD and HIV

**Table 5:** Some important liquid-crystal hydrogel formulations for vaginal drug delivery.

Formulation	Composition	Drug	Disease
Liquid-crystal precursor	Carbopol 974P, polycarbophil, water	*Syngonanthus nitens*	VVD with *Candida krusei*
Liquid-crystal precursor	Carbopol 974P, polycarbophil, water	*Syngonanthus nitens*	VVD with *Candida albicans*
Liquid-crystal gel based on phytantriol	Phytantriol, ethanol, water	Sinomenine hydrochloride	Cervical cancer
Liquid-crystal gel	Pluronic P407	Hypericin	BV, cervical and vaginal cancers

**Table 6:** Various types of mucoadhesive formulations for VDDS [53,60,61,62,63,64,65,66,67,68,69,70,71,72,73,74,75,76].

Formulation Type	Polymer	Drug	Infection
Conventional liposomes	Phosphatidylcholine and hydrogenated phosphatidylcholine	azithromycin	*E. coli* related vaginal infection
Liposomes	Chitosan and phosphatidylcholine	Resveratrol	Vaginal inflammation and infection
Liposomes	Phosphatidylcholine, monoacyl phosphatidylcholine, and propylene glycol	azithromycin	*E. coli* related vaginal infections
Liposomes (mucus penetrating)	Cholesterol, phosphatidylcholine, methoxy poly(ethylene glycol)-modified lipids	azithromycin	Vaginal candidiasis
Nanoparticles	Poly(lactic-co-glycolic acid)	Clotrimazole	*Candida albicans*
Nanoparticle	Chitosan	Miconazole	Vaginal candidiasis
Nanoparticle	Eudragit RL100 and hyaluronic acid	Ampicillin	Vaginal candidiasis
Nanocapsules	Chitosan and lecithin	Tioconazole, Econazole	Vaginal candidiasis
Mucoadhesive Liposomal gel	Phosphatidylcholine, cholesterol, pectin, and dimethyldidodecyl ammonium bromide	Sertaconazole	Vaginal candidiasis
Lipogel	Carbopol, hydroxy propyl methyl cellulose	Benzydamide hydrochloride	Vaginitis
Microgel	Polycarbophil	Miconazole nitrate	Vaginal candidiasis
Nanofibers	Polyvinyl pyrrolidone	Metronidazole	Vaginal candidiasis
Nanofibers	Dextran, sodium alginate, PVA	Clotrimazole	Vaginal candidiasis
Film/membrane	Chitosan and poly(2-ethyl-2-oxazoline)	Ciprofloxacin	Bacterial vaginosis
Film/membrane	Alginate, chitosan	Metronidazole	Bacterial vaginosis
Film/membrane	Modified gellan gum	Metronidazole	Bacterial vaginosis

## Data Availability

Not applicable.
